# Diabetes Medication Market: Costs and Utilization of Glucagon‐Like Peptide 1 (GLP‐1) Prescriptions From 2008 to 2023

**DOI:** 10.1111/1753-0407.70278

**Published:** 2026-10-08

**Authors:** Kaili Mauricio, Brielle Ruscitti, Andrew White, Diana Bowser

**Affiliations:** ^1^ William F. Connell School of Nursing Boston College Chestnut Hill Massachusetts USA; ^2^ Morrissey College of Arts and Sciences Boston College Chestnut Hill Massachusetts USA

## Abstract

The figure shows the market share of prescriptions filled from 2008 to 2023. Over this period, the GLP‐1 prescription market shifted from predominantly Byetta to a broader distribution across Victoza, Trulicity, and Ozempic, with smaller shares of Rybelsus and Mounjaro by 2023.

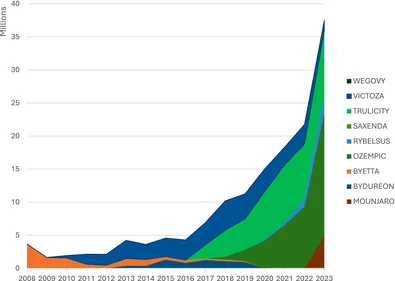

1

Type‐II diabetes mellitus (T2DM) affects a large and growing share of the U.S. population [1, 2], resulting in lower quality of life, increased likelihood of serious complications, risks for additional chronic conditions [3, 4], and significant costs to individuals and the healthcare system [5]. In 2022, the estimated medical and lost productivity costs of diagnosed T2DM were $413 billion [6].

Metformin has long served as a first‐line therapy, but glucagon‐like peptide‐1 (GLP‐1) medications have gained traction due to their effectiveness in glucose management as well as weight loss and reduced cardiovascular risk. This study examines trends in market concentration, pharmaceutical spending, and broader healthcare expenditures for GLP‐1s and metformin between 2008 and 2023.

A descriptive cost analysis was conducted using prescription and expenditure data from the Medical Expenditure Panel Survey (MEPS). The analysis covered four payer categories (Medicare, Medicaid, private insurance, and self‐pay) over the 15‐year study period. GLP‐1 market concentration was examined by brand name over time, captured by dividing the total number of GLP‐1 prescriptions within each pharmacological class by the total number of GLP‐1 prescriptions across all pharmacological classes in each year (Figure 1). The historical payer market concentration of 10 pharmaceutical classes prescribed for any individuals with a reported ICD‐10 diagnosis of diabetes was examined from 2008 to 2023 for private insurance, Medicare, and Medicaid payers. Distribution was calculated by dividing the total expenditure for GLP‐1 medications by the amount paid for by private insurance, Medicare, Medicaid, self‐pay, and other payers. Additionally, expenditures, including prescription, other healthcare, and total healthcare expenditures for diabetic patients who were prescribed either GLP‐1s or Metformin, were analyzed.

**FIGURE 1:**
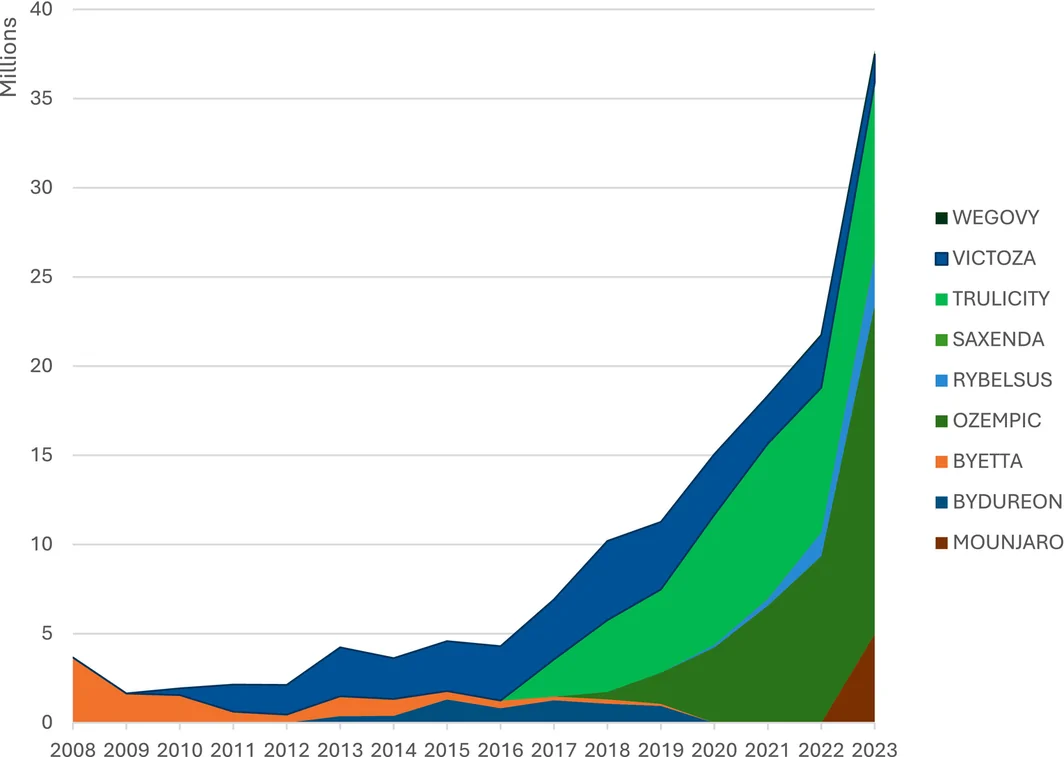
Estimated Prescription Fills of GLP‐1 Agonists by Pharmaceutical Brand, 2008–2023. *Source:* Author's analysis of MEPS Prescribed Medicines files, 2007–2023, restricted to adults with diagnosed diabetes. Estimates reflect weighted prescription fills, not unique patients; 2012 data unavailable.

Figure 1 shows shifting GLP‐1 usage over time, with Byetta and Victoza comprising most of the market (89%) until 2013; however, their market concentration falls to 46% in 2018. By 2023, following the introduction of Trulicity (2016) and Ozempic (2017), these prescriptions hold most of the market (81%). GLP‐1 market concentration grew substantially across all payer types (700% for private insurance, 750% for Medicare, and 5,006% for Medicaid). Private insurance (65%) and Medicare (45%) accounted for the largest shares of GLP‐1 spending in 2023.

Despite high prescription costs, recent studies suggest that GLP‐1 prescriptions are effective in improving health outcomes across user classifications and may act as a key preventative measure for conditions not initially considered. While GLP‐1s were considerably more expensive at $7185 per patient than Metformin annually in 2023, the results above point to descriptive trends worth noting: GLP‐1 users experienced a 3.8% reduction in other healthcare spending per person, compared to a 1.3% reduction for Metformin users. As demand for GLP‐1s continues to grow amid active price‐cap negotiations and evolving coverage policy, these findings have practical relevance for both clinicians and policymakers concerned with affordability and access. Further examination of markets and expenditures is necessary because, as of July 1, 2026, Medicare initiated the Medicare Bridge Program, which is a pilot program enabling eligible beneficiaries to access specific GLP‐1 medications [7].

## Funding

The authors have nothing to report.

## Conflicts of Interest

The authors declare no conflicts of interest.

## Data Availability

The data that support the findings of this study are available in MEPS Dataset at https://meps.ahrq.gov/mepsweb/.
